# Highly Pathogenic Avian Influenza A(H5N1) Clade 2.3.4.4b Virus in Domestic Cat, France, 2022

**DOI:** 10.3201/eid2908.230188

**Published:** 2023-08

**Authors:** François-Xavier Briand, Florent Souchaud, Isabelle Pierre, Véronique Beven, Edouard Hirchaud, Fabrice Hérault, René Planel, Angélina Rigaudeau, Sibylle Bernard-Stoecklin, Sylvie Van der Werf, Bruno Lina, Guillaume Gerbier, Nicolas Eterradossi, Audrey Schmitz, Eric Niqueux, Béatrice Grasland

**Affiliations:** ANSES, Ploufragan, France (F.-X. Briand, F. Souchaud, I. Pierre, V. Beven, E. Hirchaud, N. Eterradossi, A. Schmitz, E. Niqueux, B. Grasland);; Clinique Vétérinaire des Deux Rivières, Mauléon, France (F. Hérault);; Clinique Vétérinaire Filiavet, Bressuire, France (R. Planel);; Resalab Ouest site de Labovet Analyse, Les Herbiers, France (A. Rigaudeau);; Santé publique France, Saint-Maurice, France (S. Bernard-Stoecklin);; Université Paris Cité Institut Pasteur National Reference Center, Paris, France (S. Van der Werf);; National Reference Center for Respiratory Viruses, Lyon, France (B. Lina);; Université de Lyon, Lyon (B. Lina);; French Ministry of Food and Agriculture, Paris (G. Gerbier)

**Keywords:** influenza, highly pathogenic avian influenza virus, H5N1, clade 2.3.4.4b, cats, ducks, respiratory infections, viruses, zoonoses, France

## Abstract

We detected highly pathogenic avian influenza A(H5N1) clade 2.3.4.4b virus in a domestic cat that lived near a duck farm infected by a closely related virus in France during December 2022. Enhanced surveillance of symptomatic domestic carnivores in contact with infected birds is recommended to prevent further spread to mammals and humans.

On December 27, 2022, the avian influenza National Reference Laboratory of the Agency for Food, Environmental and Occupational Health & Safety in France confirmed a case of highly pathogenic avian influenza (HPAI) A(H5N1) clade 2.3.4.4b virus in a domestic cat. The cat lived with a human family next to a breeding duck farm, which had notified the animal health services of possible HPAI on December 9, 2022, after observing a 20% drop in egg production. After HPAI H5N1 clade 2.3.4.4b virus was confirmed at the farm, 8,375 ducks were culled on December 14. On December 20, the cat displayed disturbances in general condition, including apathy and mild hyperthermia, and was seen by a veterinary surgeon. The cat's condition worsened; pronounced neurologic and respiratory (dyspnea) symptoms appeared, resulting in compassionate euthanasia on December 24. Veterinarians collected sinonasal, tracheal, and anal swab samples after death, and a screening laboratory performed real-time reverse transcription PCR (RT-PCR) targeting the matrix protein and hemagglutinin (H5) genes. The laboratory sent H5-positive tracheal and sinonasal swab samples to the National Reference Laboratory, which confirmed HPAI H5N1 virus by using specific real-time RT-PCR for H5 clade 2.3.4.4b and neuraminidase (N1) genes (Table).

**Table:** Real-time reverse transcription PCR results for different clinical samples in study of highly pathogenic avian influenza A(H5N1) clade 2.3.4.4b virus in domestic cat, France, 2022*

Sample	PCR target gene			
	Matrix protein	H5 hemagglutinin	H5 hemagglutinin 2.3.4.4b	N1 neuraminidase
Tracheal swab	25.5	25.5	26	29.4
Sinonasal swab	33.3	31.5	32.9	36.7
Anal swab	ND	ND	NA	NA

*Values are PCR cycle thresholds. NA, not applicable; ND, not detected.

We compared the complete sequence of the HPAI H5N1 virus found in the cat (A/cat/France/22P026544/2022) with other HPAI H5N1 virus sequences circulating in France in the same area, including the virus found in the neighboring duck farm (A/duck/France/22P025647/2022). Phylogenetic analyses of HPAI H5N1 genomes indicated that the virus from the cat belonged to the A/duck/Saratov/29-02/2021–like genotype, which has been the predominant virus genotype circulating in France and Europe since September 2022. The cat virus sequence was directly related to virus sequences identified in the same area in December 2022 (Figure). Furthermore, the virus isolated from the neighboring duck farm (Figure) had only 2 nt differences (out of 13,507 total nts) compared with the cat virus, resulting in an E627K mutation in polymerase basic protein 2 and E26G mutation in nonstructural protein 2 in the cat virus. The E627K mutation has been described as a major marker of influenza virus adaptation to mammalian hosts (*1*). The E26G mutation has a possible role in virus adaptation to temperature changes (*2*). Since September 2021, a total of 90 sequences of HPAI H5N1 clade 2.3.4.4b viruses detected in mammals have been available in the GISAID EpiFlu database (https://www.gisaid.org), 20 of which have the E627K mutation, most probably indicating a rapid selection of this mutation in mammalian hosts (*3*). This virulence marker is in addition to those already observed in circulating HPAI H5N1 viruses detected in birds in Europe, such as the PB1-F2 N66S mutation (*4*).

**Figure:**
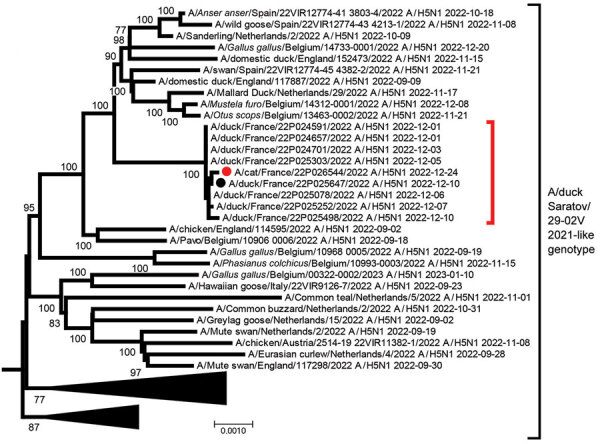
Phylogenetic analysis of highly pathogenic avian influenza A(H5N1) clade 2.3.4.4b virus detected in domestic cat, France, 2022. Tree was created by using MEGA 7 software (https://megasoftware.net) and the neighbor-joining method with 1,000 bootstrap replicates for complete concatenated HPAI H5N1 virus segments. All sequences belong to the A/duck/Saratov/29-02V/2021–like genotype. Red solid circle indicates virus sequence from cat; black solid circle indicates sequence from a nearby duck farm. Both sequences are available in the GISAID database (https://www.gisaid.org) under accession nos. EPI_ISL_16395206 (cat) and EPI_ISL_16740903 (duck). Red bracket indicates closely related sequences detected during the same period and area in France from domestic bird farms. Scale bar indicates nucleotide substitutions per site.

Since winter 2021–22, the number of reported cases of HPAI H5N1 clade 2.3.4.4b infections in mammals has increased (*5*,*6*), likely caused by several factors. First, a higher prevalence of HPAI H5 viruses in wild and domestic birds might increase the probability of interactions between infected birds and mammals (scavenging, shared habitat). Second, increased surveillance of avian influenza in wildlife might lead to more detection in mammals. Third, currently circulating viruses might infect mammalian hosts more easily. HPAI H5N1 virus detection in mammals is often linked to clinical signs, such as neurologic symptoms, or death of the animal (*3*,*7*). Few indications of intermammal HPAI H5N1 clade 2.3.4.4b contamination exist with the exception of massive infections in seal colonies in the United States (*6*) and a mink farm in Spain in 2022 (*6*,*7*). In our case report, negative results from serologic and real-time RT-PCR analyses of samples from the dog and other cat in the same household indicate a lack of intermammal transmission.

In conclusion, we show that HPAI H5N1 clade 2.3.4.4b can infect cats; HPAI H5N1 clade 1 and clade 2.2 have been sporadically detected in cats since 2004 (*8*). The close interactions and proximity of domestic cats and humans and rapid selection of mutations (after 1 passage from bird to mammal) could result in a virus with potential for interhuman transmission, indicating a considerable public health threat. Given that HPAI H5N1 circulates at high levels in wild and domestic birds, and virus was detected in a domestic cat, we recommend enhanced surveillance of symptomatic domestic carnivores in contact with infected birds to rapidly identify potential transmission events to other domestic animals and prevent further spread to humans. Our report also indicates that adequate protective equipment and barrier measures should be provided to avoid direct transmission of HPAI to persons exposed to infected birds (*6*,*9*).

Appendix Additional information for highly pathogenic avian influenza A(H5N1) clade 2.3.4.4b virus in domestic cat, France, 2022.
